# Rare case of upper gastrointestinal bleeding: Dieulafoy’ s lesion of duodenum. A case report

**DOI:** 10.1016/j.amsu.2019.07.022

**Published:** 2019-07-13

**Authors:** Pessia Beatrice, Romano Lucia, Giuliani Antonio, Gizzonio Domenico, Schietroma Mario, Carlei Francesco, Pietroletti Renato

**Affiliations:** a Department of General Surgery. Department of Biotechnological and Applied Clinical Sciences, University of L'Aquila, L'Aquila, Italy; b Department of Surgery, Val Vibrata Hospital, Teramo, Italy

**Keywords:** Dieulafoy's lesion, Upper gastrointestinal bleeding, Emergency surgery

## Abstract

Dieulafoy's lesion is an uncommon but important cause of recurrent upper gastrointestinal bleeding. Extragastric location of Dieulafoy's lesion is rare. We report a case of haemorrhagic shock due to delafoy ulcer of the duodenum. After the failure of endoscopic haemostatic treatment, and due to the hemodynamic instability, the patient was not a candidate for transarterial embolization, the emergency surgery was needed to stop the bleeding. Using minimally invasive technique, we were able to avoid radical surgery, in critical patient would have high mortality rate. Aggressive endoscopic examinations combined with the accurate treatment endoscopic, or after the failure of this, in cases of severe hemorrhagic shock, surgical approach should be adopted when Dieulafoy-like lesion is suspected as a possible cause of upper GI haemorrhage.

## Highlights

• Dieulafoy's lesion is an uncommon but important cause of recurrent upper gastrointestinal bleeding.
• Endoscopic vision shows actively bleeding, typically from point source on GI mucosa.
• The localization of Dieulafoy's lesion in the duodenum or in the small intestine can be more difficult.
• Endoscopic treatment is the first choice, often effective, repeatable, but related to a percentage of failure of 10%.
• Surgical management is indicated when the lesion fails to respond to therapeutic endoscopy.

## 1 Introduction

Dieulafoy's lesion (DL) (exulceratio simplex, cirsoid aneurysm or caliber-persistent submucosal vessel) is an unusual but important and potentially life threatening cause of gastrointestinal bleeding responsible for up to 5% of acute upper gastrointestinal bleeds [[1], [2], [3]]. The ulcer is localized typically within 6–10 cm from the gastroduodenal junction. Frequently they find themselves on the small curvature and in the gastric body (67%), less on the bottom (25%). However they have been identified lesions throughout the gastrointestinal tract from the esophagus to the anal canal [2,4], and in the bronchus [6]. The duodenum is the second most common site for a Dieulafoy's lesion (14–18% of cases) and the majority of these lesions are located in the duodenal bulb (53%) followed by the third part of the duodenum (29%) and the junction of the first and second parts of the duodenum (18%) [2,5,6]. The endoscopical appearance and its management have been discussed in this report. This work has been reported in line with the SCARE criteria [7].

## 2 Case report

A 73-year-old male, hospitalized at our department of Surgery, with a story of chronic obstruction pulmonary disease, hypertension, and end-stage renal disease, presents in week before intermittent episodes of melena. There was no history of acid peptic disease, non-steroid anti-inflammatory drugs intake, chronic liver disease, or antiplatelet or anticoagulant drugs. He arrived at emergency room with hypotension, severe pallor and tachycardia, and an important hematemesis. His laboratory exams were: hemoglobin 5.5 g/dL, Hematocrit 22%, Other hematological and biochemical investigations were within normal limits. After resuscitation therapy with fluid, plasma and blood infusion, he underwent an esophagogastroduodenoscopy (EGDS) that revealed an important actively bleeding in the duodenal bulb. After rinsing and aspiration, it is identified the source of bleeding, which was a pulsatile lesion of a few millimeters which emerged the mucosa, with no signs of local inflammation, or peptic lesions like. We performed an epinephrine injection and electrocautery, but the bleeding was not controlled. Therefore, we used the hemoclips, but the important bleeding did not permit the control of hemostasis. Due to hemodynamic instability, it was not indicated to perform transarterial embolization.

**Fig. 1:**
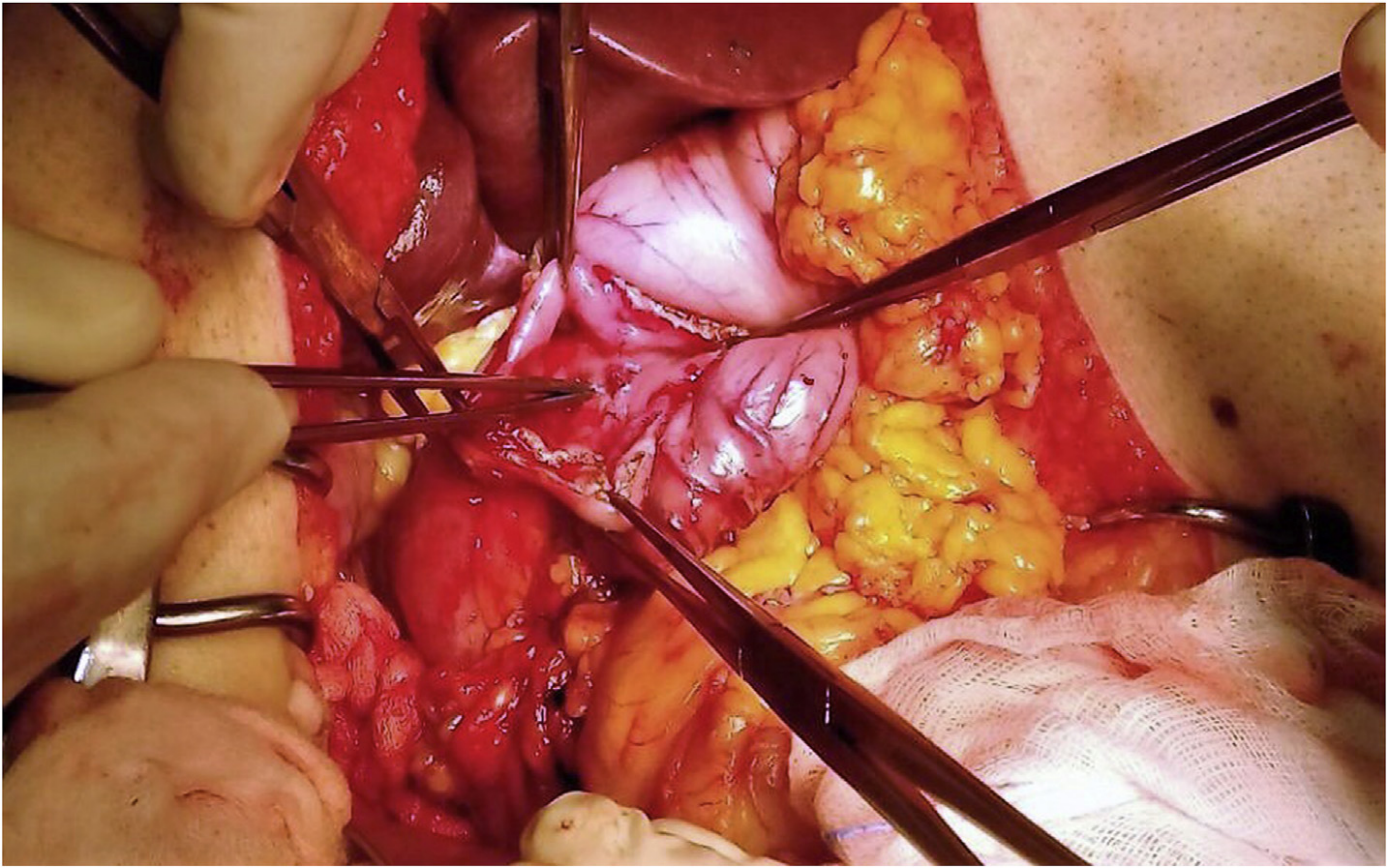
Duodenotomy: pulsatile arteriole rises of mucosal, and it opened in the lumen of duodenal bulb.

Therefore, the patient was taken to the operating room and emergency laparotomy was performed (D.G.). After mobilization of pancreatoduodenal block, we performed a longitudinal duodenotomy, we found in the duodenal bulb, large pulsatile arteriole, that rises of mucosal, and it opened in the intestinal lumen. The rest the mucosa of the duodenum was normal explored. The characteristics of the lesion were suggestive of duodenal ulcer of Dieulafoy (Fig. 1). Hemostasis was controlled with vessel ligation, and a resection was not necessary. They are needed other infusions of blood after surgery 48 h after surgery, hemoglobin was 10.2, hematocrit was 29%. He patient had no bleeding late, or complications in the postoperative period. He was discharged 8 days after surgery.

## 3 Discussion

It has been demonstrated that this injury is caused by an abnormality of anatomical arteriole under the mucosa, which appears dilated and tortuous. It starts very close to the surface of the basal membrane of the mucosa. The chronic trauma of arteriole pulse generates mucosal erosions and vessel rupture in the gastrointestinal lumen [1]. Usually the arteriole has a gauge including 1–3 mm. The lesion macroscopically has a diameter of between 2 and 5 mm. Histologically, the mucosa does not present alterations that deepen in muscularis, there are no signs of inflammation or injury of acid-related. The age-related atrophy of the gastrointestinal mucosa of the pieces can be a predisposing factor in the development of Dieulafoy ulcer, there are contrary opinions agree on the role of alcohol and the use of NSAIDs in the pathogenesis of this lesion [6,8].

Patients are typically asymptomatic before presenting with acute, profuse GI bleeding, which can manifest as hematemesis, melena, or hematochezia, however, high percentage of patients present both hematemesis and melena as in our experience. The bleeding usually is severe, but rarely patients present with chronic, occult, GI bleeding [2,[5], [6], [7], [8], [9], [10]].

The first choice, for acute GI bleeding is EGD, and DL is usually diagnosed by an endoscopic exam, which reveals a pigmented protuberance from vessel stump, with minimal surrounding erosion and no ulceration. Usually it is 10–15 mm wide and about 5–10 mm high. Endoscopic vision shows actively bleeding, typically with spurting or oozing of blood from miniscule point source on GI mucosa [5,6]. Often DL have a fresh adherent clot, and the lesion may be exposed by washing away an adherent clot with moderate endoscopic perfusion, however the authors not recommend guillotining an adherent clot covering for the risk of inducing severe hemorrhage [10]. Supplemental diagnostic methods may be endoscopic ultrasound. Typically, endosonographic features include an abnormally large caliber, pulsatile, high-flow, submucosal artery. This approach has been used to confirm endoscopic hemostasis of a bleeding DL, by demonstrating absent blood flow after therapy. However, it's more expensive, and is not currently not recommended for routine clinical practice due to insufficient data concerning efficacy [11]. The localization of Dieulafoy's lesion in the duodenum or in the small intestine can be more difficult, and in 18% of cases the diagnosis was performed after laparotomy. Endoscopic hemostatic therapy includes monotherapy with an injection of epinephrine, sclerosant, alcohol, glue or hypertonic saline or the use of thermal probe monotherapy. Other endoscopic hemostatic measures include mechanical hemostasis using bands or clips [12].

Endoscopic treatment is the first choice, often effective, repeatable, but related to a percentage of failure of 10% [[13], [14], [15]].

The mechanical therapy is better than the injection, as Chung et al. [16] have demonstrated, in fact in their study the rate of rebleeding was significantly lower in the mechanical treatment group than in the injection treatment group (8% vs. 33%).

The rate of rebleeding is lowered more when the injection therapy was combined with mechanical therapy. However, the electro and thermocoagulation methods carry a risk of transmural injury in thin walled organs like duodenum and are better suited for gastric DL. The normal surrounding mucosa in a duodenum lesions makes it an adequate target for emoclipping [13,17].

As in our case repeated endoscopy is usually necessary, and surgical management is indicated when the lesion fails to respond to therapeutic endoscopy. Surgical options include vessel ligation antroduodenectomy, or pancreas sparing duodenectomy. Although indications for emergency resections as for benign pathology have declined, this procedure is still necessary as a salvage option when conservative management has failed or is not practicable [17].

## 4 Conclusion

Endoscopic diagnosis of extragastric Dieulafoy's lesion can be difficult because of the small size and obscure location of the lesion. Increased awareness and careful and early endoscopic evaluation following the bleeding episode are the key to accurate diagnosis. The endoscopic modalities for control of bleeding is the first choice of treatment of the lesion, but often the hemostasis is not possible. Emergency surgery after the failure of 'surgical endoscopy, and haemodynamic instability status, is the only choice to solve the bleeding. However, in our case the hemostatic ligation of the bleeding vessel allowed to stop the bleeding and avoid a debulking surgery in critically ill patients.

## Conflicts of interest

No conflict of interest.

## Sources of funding

No funding.

## Ethical approval

Ethical approval was not required.

## Author contribution

Pessia Beatrice, Giuliani Antonio: Writing.

Romano Lucia, Gizzonio Domenico: Data collection.

Mario Schietroma, Francesco Carlei, Pietroletti Renato: Study design and review.

## Guarantor

The Guarantor is the one or more people who accept full responsibility for the work and/or the conduct of the study, had access to the data, and controlled the decision to publish.

Prof. Francesco Carlei.

## Consent

Written informed consent was obtained from the patient for publication of this case report and accompanying images. A copy of the written consent is available for review by the Editor-in-Chief of this journal on request.

## Conflicts of interest and source of funding

The authors declare that they have no conflict of interest.

No funding.

## Provenance and peer review

Not commissioned, internally reviewed.

## References

[1] Sciumè C., Di Vita G., Geraci G., Pisello F., Modica G. (2001). Dieulafoy's ulcer: unusual cause of upper gastrointestinal tract. Our experience. Ann. Ital. Chir..

[2] Schietroma M., Cecilia E.M., De Santis G., Carlei F., Pessia B. (2016 Feb). Amicucci G peri-operative oxygen and incision site infection after surgery for perforated peptic ulcer: a randomized, double-blind monocentric trial. Surg. Infect..

[3] Sista F., Abruzzese V.1, Schietroma M. (2013). Concomitant gastrointestinal stromal tumor of the stomach and gastric adenocarcinoma in a patient with billroth 2 resection. Case Rep Surg.

[4] Cortese F., Colozzi S., Marcello R., Muttillo I.A., Giacovazzo F., Nardi M., Mero A. (2013). Gastroduodenal major haemorrhages in critical patients: an original surgical technique. Ann. Ital. Chir..

[5] Chaer R.A.1, Helton W.S. (2003). Dieulafoy's disease. J. Am. Coll. Surg..

[6] Rana S., Bhasin D.K., Gupta R., Yadav T.D., Gupta V., Singh (2010). Periampullary Dieulafoy's lesion: an unusual cause of gastrointestinal bleeding. JOP.

[7] Agha R.A., Borrelli M.R., Farwana R., Koshy K., Fowler A., Orgill D.P. (2018). For the SCARE group. The SCARE 2018 statement: updating consensus surgical CAse REport (SCARE) guidelines. Int. J. Surg..

[8] Giuliani A., Romano L., Papale E. (2019 Feb 13). Complications post-laparoscopic sleeve gastric resection: review of surgical technique. Minerva Chir..

[9] Veldhuyzen Van Zanten S.J.O., Bartelsman J.F., Schipper M.E., Tytgat G.N.J. (1986). Recurrent massive haematemesis from Dieulafoy vascular malformations-a review of 101 cases. Gut.

[10] Nojkov B., Cappell M.S. (2015). Gastrointestinal bleeding from Dieulafoy's lesion: clinical presentation, endoscopic findings, and endoscopic therapy. World J. Gastrointest. Endosc..

[11] Lim W., Kim T.O., Park S.B., Rhee H.R., Park J.H., Bae J.H. (2009). Endoscopic treatment of Dieulafoy lesions and risk factors for bleeding. Korean J. Intern. Med..

[12] Jaspersen D. (1993). Dieulafoy's disease controlled by Doppler ultrasound endoscopic treatment. Gut.

[13] Sone Y., Kumada T., Toyoda H., Hisanaga Y., Kiriyama S., Tanikawa M. (2005). Endoscopic management and follow up of Dieulafoy lesion in the upper gastrointestinal tract. Endoscopy.

[14] Fujishiro M., Iguchi M., Kakushima N. (2016). Guidelines for endoscopic managements of non-variceal upper gastrointestinal bleeding. Dig. Endosc..

[15] Marchese M., Romano L., Giuliani A. (2019). Corrigendum to "A case of intrasplenic displacement of an endoscopic double-pigtailstent as a treatment for laparoscopic sleeve gastrectomy leak" [Int. J. Surg. Case Rep. 53 (2018) 367-369]. Int J Surg Case Rep.

[16] Chung I.K., Kim E.J., Lee M.S. (2000). Bleeding Dieulafoy's lesions and the choice of endoscopic method: comparing the hemostatic efficacy of mechanical and injection methods. Gastrointest. Endosc..

[17] Chereau N., Chandeze M.M., Tantardini C. (2016). Antroduodenectomy with gastroduodenal anastomosis: salvage emergency surgery for complicated peptic ulcer disease-results of a double institution study of 35 patients. J. Gastrointest. Surg..

